# Camrelizumab plus platinum-irinotecan followed by maintenance camrelizumab plus apatinib in untreated extensive-stage small-cell lung cancer: a nonrandomized clinical trial

**DOI:** 10.3389/fimmu.2023.1168879

**Published:** 2023-04-11

**Authors:** Jun Ni, Xiaoyan Si, Hanping Wang, Xiaotong Zhang, Li Zhang

**Affiliations:** ^1^ Department of Pulmonary and Critical Care Medicine, Peking Union Medical College Hospital, Chinese Academy of Medicine Sciences & Peking Union Medical College, Beijing, China; ^2^ Department of Respiratory and Critical Care Medicine, Beijing Chao-Yang Hospital, Capital Medical University, Beijing, China

**Keywords:** small cell lung carcinoma (SCLC), platinum, irinotecan, camrelizumab, immune checkpoint inhibitors

## Abstract

**Background:**

Programmed cell death-ligand 1 (PD-L1) inhibitors plus chemotherapy have made substantial progress in extensive-stage small-cell lung cancer (ES-SCLC), but the survival benefit is still limited. This study aimed to evaluate the preliminary efficacy and safety of camrelizumab plus platinum-irinotecan (IP/IC) followed by maintenance camrelizumab plus apatinib in patients with untreated ES-SCLC.

**Methods:**

In this non-randomized clinical trial (NCT04453930), eligible patients with untreated ES-SCLC received 4-6 cycles of camrelizumab plus IP/IC, followed by maintenance with camrelizumab plus apatinib until disease progression or unmanageable toxicity. The primary endpoint was progression-free survival (PFS). Patients who received PD-L1 inhibitors (atezolizumab or durvalumab) plus platinum-etoposide (EP/EC) were selected as the historical control.

**Results:**

Nineteen patients received IP/IC plus camrelizumab and 34 patients received EP/EC plus PD-L1 inhibitor. At a median follow-up time of 12.1 months, the median PFS was 10.25 months (95% CI: 9.40-NA) in the IP/IC plus camrelizumab group and 7.10 months (95% CI 5.79-8.40) in the EP/EC plus PD-L1 inhibitor group, respectively (HR=0.58, 95% CI 0.42-0.81). The objective response rate of IP/IC plus camrelizumab and EP/EC plus PD-L1 inhibitor was 89.6% and 82.4%, respectively. The most common treatment-related adverse events in the IP/IC plus camrelizumab group was neutropenia, followed by reactive cutaneous capillary endothelial proliferation (RCCEP) and diarrhea. The occurrence of immune-related adverse event was found to be associated with a prolonged PFS (HR=4.64, 95% CI 1.92-11.18).

**Conclusions:**

IP/IC plus camrelizumab followed by maintenance camrelizumab plus apatinib showed preliminary efficacy and acceptable safety profile in patients with untreated ES-SCLC.

## Introduction

Approximately 15% of lung cancer cases are small-cell lung cancer (SCLC), which has a poor prognosis (1). Platinum-based chemotherapy has been the standard first-line treatment for both limited-stage (LS) and extensive-stage (ES) disease since the 1980s, with an objective response rate (ORR) of about 65% and a one-year survival rate of 30%-40% for ES-SCLC (2, 3).

Since the advent of immune checkpoint inhibitors (ICIs), treatment for many solid tumors has been subverted. Furthermore, SCLC has a high mutation rate, making ICIs an attractive therapeutic option (4). Two phase 3 trials evaluated the addition of programmed cell death-ligand 1 (PD-L1) inhibitor atezolizumab (IMpower133) (5, 6) or durvalumab (CASPIAN) (7, 8) to first-line chemotherapy (etoposide and cisplatin [EP]) followed by maintenance PD-L1 inhibitor in patients with ES-SCLC, and showed a significant improvement in overall survival (OS). Based on the above two studies, PD-L1 inhibitor combined with chemotherapy followed by maintenance PD-L1 inhibitor has been the standard care for untreated ES-SCLC now. However, compared with the unprecedented success of PD-L1 combined with EP in the first-line treatment of ES-SCLC patients, programmed cell death 1 (PD-1) inhibitors showed unsatisfactory efficacy outcomes. KEYNOTE-604 study reported an improvement in progression-free survival (PFS) but not in OS with first-line pembrolizumab combined with EP in patients with ES-SCLC (9). Other therapeutic regimens with PD-1 inhibitors therefore remain to be explored.

Camrelizumab is a humanized high-affinity IgG4-kappa anti-PD-1 monoclonal antibody, which has been approved in combination with chemotherapy (carboplatin plus paclitaxel or pemetrexed) for the first-line treatment of advanced non-small-cell lung cancer (NSCLC) (10, 11). However, the efficacy and safety of camrelizumab plus chemotherapy for ES-SCLC patients remains unknown. In patients with ES-SCLC, two of the most commonly used first-line chemotherapy regimens are platinum (cisplatin or carboplatin) combined with etoposide (EP/EC) or irinotecan (IP/IC). A phase III trial conducted in Japan demonstrated a survival benefit of IP regimen over EP regimen for previously untreated ES-SCLC (12). Moreover, the PASSION study found that the ORR of apatinib (an antiangiogenic agent) combined with camrelizumab was 34.0%, with a median PFS of 3.6 months and a median OS of 8.4 months in patients with ES-SCLC who failed platinum-based chemotherapy (13). Thus, based on the potential therapeutic effect of IP in Asian patients and the promising efficacy of camrelizumab plus apatinib as second-line regimen for ES-SCLC, this non-randomized trial was conducted to explore the preliminary efficacy and safety of camrelizumab plus platinum-irinotecan followed by maintenance camrelizumab plus apatinib in patients with untreated ES-SCLC, with comparison of our historical cohort who received PD-L1 inhibitors plus platinum-etoposide.

## Methods

### Study design and patients

This non-randomized clinical trial was approved by the ethics committee of Peking Union Medical College Hospital, and was registered with ClinicalTrials.gov (NCT04453930). All patients signed the informed consent before any procedure. The key inclusion criteria were patients aged 18 to 75 years old; pathologically or cytologically confirmed ES-SCLC; previously untreated (including radiotherapy, chemotherapy, vascular endothelial growth factor receptor [VEGFR] inhibitors and ICIs); with an Eastern Cooperative Oncology Group performance status (ECOG PS) score of 0-1. Patients with active brain metastasis or meningeal metastasis were excluded. Detailed inclusion and exclusion criteria were presented in the **Supplementary Materials**.

### Procedure

Eligible patients were administrated camrelizumab (200 mg, day 1), irinotecan (65 mg/m^2^, days 1 and 8) plus platinum (cisplatin: 30 mg/m^2^, days 1 and 8; or carboplatin: area under curve [AUC]=4~5, day 1) every 3 weeks for 4-6 cycles as induction therapy. After induction therapy, patients were assessed for efficacy per Response Evaluation Criteria in Solid Tumors version 1.1 (RECIST 1.1). If progressive disease (PD) was not occurred, patients then received maintenance therapy with camrelizumab (200 mg, day 1, every 3 weeks) and oral apatinib (250 mg, once daily) until disease progression or unacceptable toxicity. Prophylactic brain irradiation was allowed after the induction therapy.

Efficacy was assessed per RECIST 1.1 criteria after the first cycle, and every two cycles thereafter in both induction treatment phase and maintenance treatment phase. For patients without PD but who discontinued the treatment, efficacy was assessed every eight weeks until PD or death. Follow-up for survival was conducted every eight weeks until death or loss of follow-up. Adverse events (AEs) were recorded and graded according to the National Cancer Institute Common Terminology Criteria for Adverse Events (NCI CTCAE, version 5.0).

### Endpoints

The primary endpoint was PFS, which defined as the time from enrollment to PD per RECIST 1.1 criteria or death from any cause, whichever occurred first. The secondary endpoints included OS (defined as the time from enrollment to death from any cause), disease control rate (DCR, defined as the percentage of patients with a complete response [CR], partial response [PR] or stable disease [SD]), ORR (defined as the percentage of patients with a CR or PR), and duration of response (DoR, defined as the time from the first document CR or PR to PD or death from any cause). The safety profiles included treatment-related AEs (TRAEs) and immune-related AEs (irAEs).

### Historical cohort

Using the electronic medical record system of Peking Union Medical College Hospital, ES-SCLC patients who received ICIs combined with chemotherapy as the first-line treatment were retrospectively reviewed. Among them, patients who received EP/EC combined with PD-L1 blockades (atezolizumab or durvalumab) were selected as the historical control. Information of baseline characteristics, treatment pattern, efficacy and safety profiles of patients were reviewed.

### Statistical analysis

All statistical analyses were conducted using SPSS version 23.0 and RStudio 1.2.5001. Continuous variables were expressed as median and range, and classification variables were expressed as frequency and percentage. Kaplan-Meier method was used to evaluate PFS and DoR, and Cox proportional hazards model was used to estimate hazard ratio (HR) and 95% confidence interval (CI). PFS was analyzed in different subgroups according to age (≤65 years, >65 years), sex (male, female), metastatic sites (with or without brain metastasis, with or without liver metastasis), modified neutrophil to lymphocyte ratio (dNLR, ≤3, >3), serum sodium (<135, ≥135), serum lactate dehydrogenase (LDH, ≤260, >260), the absolute value of CD4+ cells (≤600/μl, >600/μl), the absolute value of CD8+ cells (≤450/μl, >450/μl), CD4+/CD8+ (1-2, <1 or >2) and the occurrence of irAEs (with or without irAEs).The HR and 95%CI of subgroups were calculated using the non-stratified Cox risk ratio model, and the association between different subgroups and median PFS was calculated. *P* < 0.05 was considered statistically significant.

## Results

### Patient baseline

From March 2020 to December 2021, 19 ES-SCLC patients were enrolled in this study and received study medications. Of them, the median age was 63 (range 51-70) years old, and 17 (89.5%) patients were male. Most patients (89.5%) were stage IV. Two patients had liver metastasis and one patient experienced brain metastasis (**Table 1**).

**Table 1:** Baseline characteristics of patients.

Characteristics	IP/IC+camrelizumab (N=19)	EP/EC+PD-L1 (N=34)	P value
Age, median (range), years	63 (51–70)	64 (42-77)	0.392
Sex (male)	17 (89.5%)	27 (79.4%)	0.324
Smoking status			0.090
Never smoked	4 (21.2%)	14 (41.2%)	
Smoking index ≥400*	11 (52.6%)	17 (50%)	
ECOG PS score			0.725
≤1	19 (100%)	32 (94.1%)	
≥2	0	2 (5.9%)	
TNM staging			0.074
Stage IIIC	2 (10.5%)	3 (8.8%)	
Stage IV	17 (89.5%)	31 (91.2%)	
Liver metastasis	2 (10.5%)	6 (17.6%)	0.497
Brain metastasis	1 (5.3%)	3 (8.8%)	0.125
Bone metastasis	5 (26.3%)	14 (41.2%)	0.288
Adrenal metastases	2 (10.5%)	4 (11.8%)	0.894
Subcutaneous muscular metastasis	0	1 (2.9%)	0.460
Paraneoplastic syndrome	1 (5.3%)	5 (14.7%)	0.307
dNLR			0.285
≤3	13 (68.4%)	28 (82.4%)	
>3	6 (31.6%)	6 (17.6%)	
Serum sodium (Na)			0.093
≥135	18 (94.7%)	26 (76.5%)	
<135	1 (5.3%)	8 (23.5%)	
Serum LDH			0.613
≤260U/L	12 (63.2%)	19 (55.9%)	
>260U/L	7 (36.8%)	15 (44.1%)	
Absolute value of CD4+ T cells			0.789
≥600/μl	13 (68.4%)	22 (64.7%)	
<600/μl	6 (31.6%)	12 (35.3%)	
Absolute value of CD8+ T cells			0.955
≤450/μl	13 (68.4%)	23 (67.6%)	
>450/μl	6 (31.6%)	11 (32.4%)	
CD4+/CD8+			0.527
1.0~2.0	10 (52.6%)	21 (61.8%)	
<1 or >2	9 (47.4%)	13 (38.2%)	
Serum ProGRP			0.641
≤3 ULN	5 (26.3%)	7 (20.6%)	
>3 ULN	14 (73.7%)	27 (79.4%)	
Serum NSE			0.327
≤3 ULN	9 (47.4%)	21 (61.8%)	
>3 ULN	10 (52.6%)	13 (38.2%)	
Number of ICI cycles, median (range)	7 (3-26)	6 (2-17)	0.051
Number of chemotherapy cycles, median (range)	6 (3-6)	5 (2-6)	0.060

EP/EC, (etoposide plus cisplatin, or etoposide plus carboplatin); IP/IC, (irinotecan plus cisplatin, or irinotecan plus carboplatin); ECOG PS, Eastern Cooperative Oncology Group performance status; dNLR, modified neutrophil to lymphocyte ratio; LDH, lactate dehydrogenase; ProGRP, pro-gastrin releasing peptide; NSE, neuron-specific enolase; ULN, upper limit of normal; ICI, immune checkpoint inhibitor.

*The smoking index was calculated as cigarettes per day x duration of smoking (years).

In addition, a total of 54 ES-SCLC patients received ICIs combined with chemotherapy as the first-line treatment were retrospective reviewed form January 2013 to December 2021, of which 34 patients treated with EP/EC combined with a PD-L1 inhibitor (atezolizumab or durvalumab) were selected as the historical control group. The median age of these patients was 64 (range 42-77) years old and 27 (79.4%) patients were male. Two patients had an ECOG PS score of 2 or above. The majority (97.1%) were stage IV. Six (17.6%) and three (8.8%) patients had liver metastasis and brain metastasis, respectively. There were no significant differences in the baseline characteristics of patients between two groups (**Table 1**; **Figure S1**).

### Efficacy profiles

**Figure 1:**
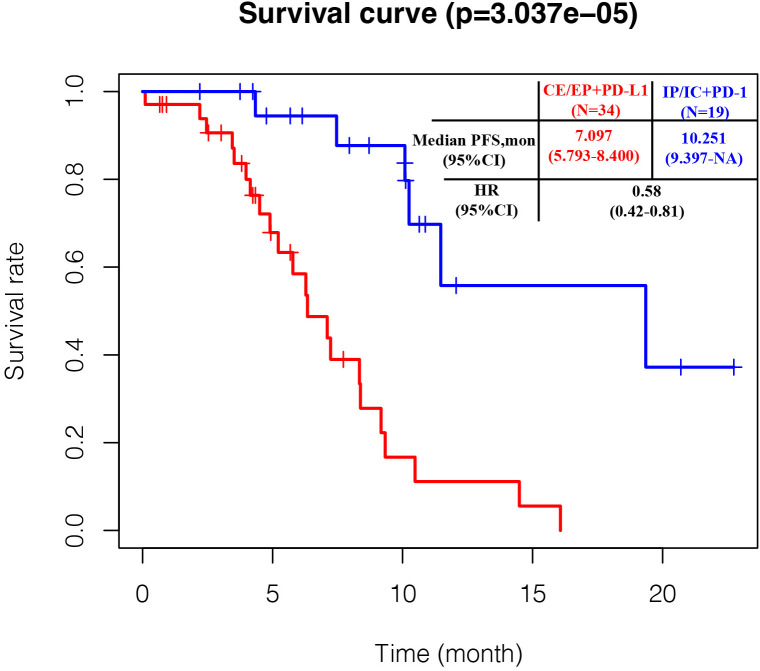
Kaplan-Meier curves of progression-free survival.

At the time of data cut-off (March 16, 2022), the median follow-up time was 12.1 months. Nine (47.4%) patients experienced PD and four (21.1%) died in the IP/IC plus camrelizumab group, with a median PFS of 10.25 (95% CI 9.40-NA) months. In the EP/EC plus PD-L1 group, 18 (52.9%) patients developed PD and six (17.6%) patients died, with a median PFS of 7.10 (95% CI 5.79-8.40) months. IP/IC plus camrelizumab showed better PFS than EP/EC plus PD-L1 as first-line treatment in patients with ES-SCLC (HR=0.58, 95% CI 0.42-0.81; *P*=0.0013) (**Figure 1**). The median OS was not achieved in both groups.

In the IP/IC plus camrelizumab group, 17 (89.5%) patients had PR, and two (10.5%) had SD, with a median DoR of 8.74 months. In the EP/EC plus PD-L1 inhibitor group, 28 (82.4%) patients had PR, four (11.8%) had SD, and two (5.9%) had PD. The median DoR was 4.4 months. The DCR and ORR were 100% and 89.6% in the IP/IC plus camrelizumab group, as well as 94.1% and 82.4% in the EP/EC plus PD-L1 inhibitor group, respectively (**Table 2**).

**Table 2:** Treatment efficacy.

Variables	IP/IC+camrelizumab (N=19)	EP/EC+PD-L1 (N=34)
PR	17 (89.5%)	28 (82.4%)
SD (≥6w)	2 (10.5%)	4 (11.8%)
PD	0	2 (5.9%)
DoR, months (95% CI)	8.74 (0.72-19.09)	4.4 (0.23-14.62)
Still response at the last follow-up	10 (52.6%)	14 (41.2%)
DCR	19 (100%)	32 (94.1%)
ORR	17 (89.6%)	28 (82.4%)
Median PFS, months (95% CI)	10.25 (9.40-NA)	7.10 (5.79-8.40)

EP/EC, (etoposide plus cisplatin, or etoposide plus carboplatin); IP/IC, (irinotecan plus cisplatin, or irinotecan plus carboplatin); PR, partial response; DoR, duration of response; SD, stable disease; PD, progression disease; DCR, disease control rate; ORR, objective response rate; PFS, progression-free survival.

As of the last follow-up, 18 (53.9%) patients with EP/EC plus PD-L1 inhibitor and nine (47.4%) patients with IP/IC plus camrelizumab developed PD. Post-progression treatment was at the discretion of the investigator depending on the patient’s condition. Of these patients with PD in the EP/EC plus PD-L1 inhibitor group, eight (23.5%) patients subsequently started receiving PD-L1 inhibitor plus other chemotherapy regimens, one (2.9%) patient received topotecan, one (2.9%) patient received albumin paclitaxel monotherapy, and eight (53.9%) patients started receiving IP/IC plus PD-1 inhibitor. In the IP/IC plus camrelizumab group, four (10.5%) patients started treatment with EP/EC plus PD-L1 inhibitor (atezolizumab/durvalumab) following progression, four (10.5%) patients began EP/EC treatment, and one patient (5.3%) received anlotinib monotherapy.

### Safety profiles

In the IP/IC plus camrelizumab group, the median cycle number was six for chemotherapy and seven for camrelizumab, respectively; while in the EP/EC plus PD-L1 inhibitor group, the median cycle number was five for chemotherapy and six for immunotherapy. The most common TRAE in the IP/IC plus camrelizumab group was neutropenia, followed by reactive cutaneous capillary endothelial proliferation (RCCEP) and diarrhea (**Table 3**). The irAEs in the IP/IC plus camrelizumab group included RCCEP (52.6%), abnormal liver function (21.1%), rash (10.5%), hypothyroidism (5.3%), oculomotor paralysis (5.3%) and kidney injury (5.3%) (**Table 4**).

**Table 3:** Treatment-related adverse events.

TRAE	IP/IC+camrelizumab (N=19)		EP/EC+PD-L1 (N=34)	
	Grade 1-2	Grade 3-4	Grade 1-2	Grade 3-4
Neutropenia	10 (52.6%)	7 (36.8%)	19 (55.9%)	9 (26.5%)
RCCEP	10 (52.6%)	0	0	0
Diarrhea	7 (36.8%)	1 (5.3%)	0	0
Nausea and vomiting	7 (36.8%)	0	11 (32.4%)	1 (2.9%)
Platelet count decreased	5 (26.3%)	2 (10.5%)	6 (17.6%)	1 (2.9%)
Hemoglobin decreased	5 (26.3%)	1 (5.3%)	10 (29.4%)	1 (2.9%)
Weakness	5 (26.3%)	0	9 (26.5%)	0
Abnormal liver function	3 (15.8%)	1 (5.3%)	0	3 (8.8%)
Alopecia	3 (15.8%)	0	3 (8.8%)	0
Rash	1 (5.3%)	1 (5.3%)	0	0
Hypothyroidism	1 (5.3%)	0	3 (8.8%)	0
Kidney injury	1 (5.3%)	0	0	0
Constipation	0	0	4 (11.8%)	0
Pneumonia	0	0	2 (5.9%)	0
Thrush	0	0	1 (2.9%)	0
Fever	0	0	1 (2.9%)	0
Myelitis	0	0	0	1 (2.9%)
Oculomotor paralysis	0	1 (5.3%)	0	0
Creatine kinase increased	0	0	1 (2.9%)	0

EP/EC, (etoposide plus cisplatin, or etoposide plus carboplatin); IP/IC, (irinotecan plus cisplatin, or irinotecan plus carboplatin); TRAE, treatment-related adverse event; RCCEP, reactive cutaneous capillary endothelial proliferation.

**Table 4:** Immune-related adverse events.

irAE	IP/IC+camrelizumab (N=19)		EP/EC+PD-L1 (N=34)	
	Grade 1-2	Grade 3-4	Grade 1-2	Grade 3-4
RCCEP	10 (52.6%)	0	0	0
Abnormal liver function	3 (15.8%)	1 (5.3%)	1 (2.9%)	2 (5.9%)
Rash	1 (5.3%)	1 (5.3%)	0	0
Oculomotor paralysis	0	1 (5.3%)	0	0
Kidney injury	1 (5.3%)	0	0	0
Hypothyroidism	1 (5.3%)	0	3 (8.8%)	0
Pneumonia	0	0	2 (5.9%)	0
Myelitis	0	0	0	1 (2.9%)
Creatine kinase increased	0	0	1 (2.9%)	0

EP/EC, (etoposide plus cisplatin, or etoposide plus carboplatin); IP/IC, (irinotecan plus cisplatin, or irinotecan plus carboplatin); irAE, immune-related adverse event; RCCEP, reactive cutaneous capillary endothelial proliferation.

### Subgroup analysis

**Figure 2:**
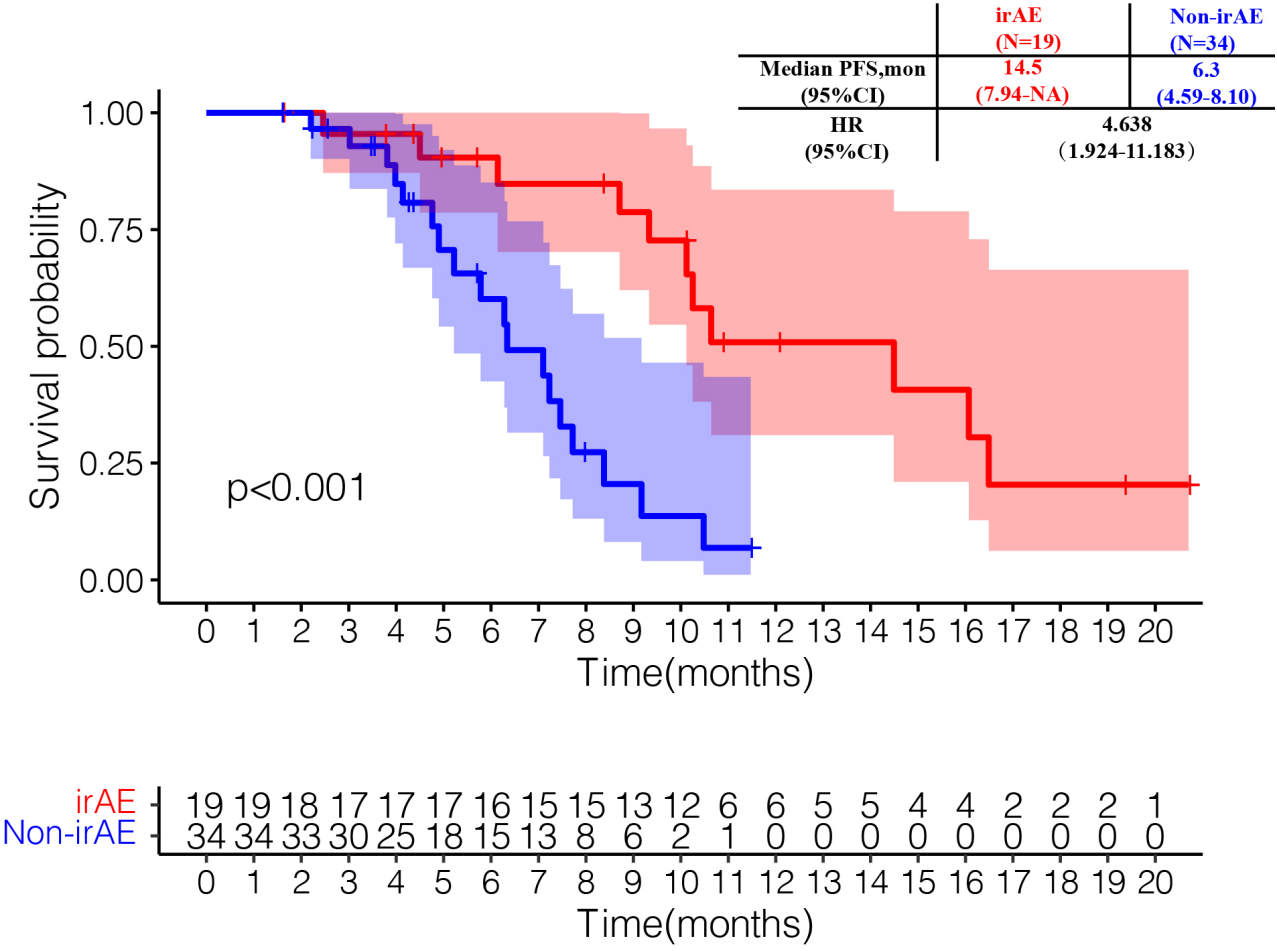
Kaplan-Meier curves of progression-free survival by immune-related adverse events (irAEs) in the irAE-evaluable population.

The IP/IC plus camrelizumab showed better PFS than EP/EC plus PD-L1 inhibitor among all subgroups, but the difference was not statistically significant (**Figure S2**). For all enrolled patients, 19 patients experienced irAEs. The median PFS in patients with irAEs was 14.5 months (95% CI 7.94-NA), which was significantly longer than that in patients without irAEs (14.5 months vs. 6.3 months, HR=4.64, 95% CI 1.92-11.18) (**Figure 2**).

## Discussion

In the past 30 years, platinum-based chemotherapy has been used as a first-line treatment for ES-SCLC patients (14, 15). Because SCLC has high tumor mutation burden, ICIs offers a new way to treat this disease and prolong patient survival. There is no doubt that PD-L1 inhibitor in combination with chemotherapy has become the standard first-line treatment for ES-SCLC patients. However, the survival benefit of ICI treatment on SCLC patients is less than that on NSCLC patients, and the OS of SCLC is still limited (4, 16). Therefore, exploring new combined treatment options is urgently needed. To our knowledge, this is the first study to report the efficacy and safety of IP/IC plus camrelizumab followed by maintenance camrelizumab plus apatinib as the first-line treatment in ES-SCLC. Compare with PD-L1 inhibitor plus EP/EC (the historical control), IP/IC plus camrelizumab had the comparable ORR (89.6% vs 82.4%), but achieved a better median PFS (10.25 months vs. 7.10 months, HR=0.58, 95% CI 0.42-0.81). The OS data was immature in both groups.

PD-1 inhibitors are able to block both PD-1/PD-L1 pathways as well as PD-1/PD-L2 pathways, whereas PD-L1 inhibitors only inhibit PD-1/PD-L1 (17). Therefore, when PD-L1 inhibitors are used, the tumor can escape through the PD-1/PD-L2 axis. Based on a meta-analysis, NSCLC patients treated with PD-1 inhibitor plus chemotherapy had a lower death risk compared to those treated with PD-L1 inhibitor plus chemotherapy (RR=0.66, 95% CI 0.48-0.90) (18). However, a different scenario was shown in untreated ES-SCLC. In the first-line treatment of ES-SCLC, IMpower133 and CASPAIN studies have demonstrated the survival benefit of PD-L1 inhibitor atezolizumab or durvalumab plus chemotherapy, while KEYNOTE-604 study of PD-1 inhibitor pembrolizumab plus chemotherapy only showed a PFS benefit but failed to show an OS benefit (5–8, 19). Nevertheless, was too early to draw a conclusion that that ES-SCLC would not benefit from the first-line therapy with PD-1 inhibitor (20). In our study, a superior PFS was also observed in the IP/IC plus camrelizumab group compared with in the EP/EC plus PD-L1 inhibitor group, whereas OS data were not yet mature at the time of data cutoff. Considering that the chemotherapy regime and maintenance therapy were distinct from the above-mentioned studies, mature OS data from our study are worthy of anticipation.

Chemotherapy and radiotherapy have the ability to increase the immunogenicity of tumor cells, increase major histocompatibility class I (MHC-I) molecule expression, activate immune effector factors such as nature killer cells, and promote immune response by targeting tumor immunosuppressive cells (21–23). In addition, radiotherapy and chemotherapy can reduce the immunosuppressive properties of tumor cells, thereby stimulating T cell activation and resulting in tumor shrinkage (24). SCLC is a disease that is highly sensitive to chemotherapy and radiotherapy. The preferred chemotherapy regimen for initial treatment of patients with ES-SCLC is EP/EC, followed by IP/IC; whereas two meta-analyses of randomized controlled trials reported a favorable OS and less hematological toxicities in irinotecan/platinum regimens compared with etoposide/platinum regimens in this population (25, 26). Therefore, in contrast to the EP/EC regimen used as a combination with immunotherapy in the IMpower133 and CASPIAN studies (5, 8), we chose IP/IC regimen when combined with camrelizumab in this study.

Maintenance PD-L1 inhibitor was administrated in IMpower133 and CASPIAN studies and pembrolizumab plus chemotherapy was continued until disease progression in KEYNOTE-604 study, whereas in our study camrelizumab combined with apatinib was used for maintenance therapy. Preclinical data have shown that apatinib could relieve hypoxia, enhance CD8+ T cell infiltration, decrease the recruitment of tumor-associated macrophages in tumors and the amount of TGF-β in both tumors and serum, and generate synergistic antitumor effects with PD-L1 blockade in lung cancer (27). Moreover, the PASSION study evaluated the efficacy of camrelizumab combined with apatinib in the treatment of recurrent ES-SCLC, and showed promising efficacy and manageable safety profile (13). These suggested this combination may have promising therapeutic effects on untreated ESCLC; however, further studies are needed to elucidate its exact molecular mechanism.

The median number of chemotherapy cycles in our study was six for IP/IC and five for EP/EC, compared to the standard four cycles. Although the latest NCCN guideline for SCLC also recommends 4 cycles of treatment for untreated ES-disease, patients may receive up to 6 cycles based on response and tolerability after 4 cycles (28). Additionally, similar overall safety profiles and grade 3-4 TRAEs were observed in two groups. The most common grade 3 or higher TRAE in both groups was neutropenia, which was reversible by symptomatic treatment (such as with granulocyte-colony stimulating factor), and its incidence rate was higher with IP/IC plus camrelizumab than with EP/EC plus PD-L1 inhibitor. Neutropenia is often induced by chemotherapy. Previous retrospective study reported that the development of severe neutropenia with IP regimen might be associated with improved prognosis as opposed to EP therapy in patients with ES-SCLC (29). In addition, RCCEP and diarrhea were the two most common TRAEs of any grade in the IP/IC plus camrelizumab group, whereas none occurred in the EP/EC plus PD-L1 inhibitor group. Diarrhea was generally reversible with dose modification or antidiarrhea therapy. Given that the vast majority of diarrhea were grade 1-2, whether prophylactic loperamide is necessary deserves further exploration. RCCEP was observed in more than half of the IP/IC combined with camrelizumab group, which may be related to the activation of vascular endothelial cell proliferation by camrelizumab through regulating vascular receptor VEGFR2, leading to vascular proliferation (30, 31). No new safety signals were observed in this study.

Notably, the subgroup analysis showed that the occurrence of irAEs was associated with a longer median PFS, which was consistent with the results of SCLC with larger samples (32), NSCLC (33), malignant melanoma (34), and metastatic renal clear cell carcinoma (35). In 2020, a multicenter retrospective study showed that the occurrence of irAEs was an independent protective factor for ES-SCLC patients treated with ICIs (PFS: HR=0.44, 95% CI 0.29-0.66; OS: HR=0.47, 95%CI 0.32-0.71) (32). Besides, it was demonstrated that the development of irAEs was associated with survival outcome in patients with advanced or recurrent NSCLC treated with nivolumab (median PFS: 9.2 months vs. 4.4 months, *P*=0.04; median OS: NA vs. 11.1 months, *P*=0.01) (33). A retrospective study of 195 NSCLC patients treated with nivolumab found that the incidence of irAEs was 43.6%, and that the ORR (43.5% vs 10%, *P*<0.001) and median PFS (5.7 months vs 2.0 months, *P*<0.001) of patients with irAEs were significantly improved (36).

This study has some limitations. First, the control group was retrospectively reviewed with selective bias, so the results of this study cannot fully reflect the efficacy and safety of the EP/EC plus PD-L1 inhibitor in patients with ES-SCLC. Second, since this study was a single-center analysis, the results might be influenced by the level of diagnosis and treatment at our center. Third, the sample size was small, and finally only 53 patients with ES-SCLC were analyzed, resulting in insufficient statistical power of our study. Last, the data of OS was immature, and the patients’ survival situation should be continuously tracked. Further large-scale randomized controlled trials are warranted to confirm our results.

## Conclusion

In conclusion, our study demonstrated the preliminary efficacy and manageable safety of IP/IC plus camrelizumab followed by maintenance camrelizumab plus apatinib in untreated ES-SCLC, compared with EP/EC plus PD-L1 inhibitor. The occurrence of irAEs was associated with a longer PFS, which may be a potential prognostic factor for patients treated with ICIs.

## Data availability statement

The original contributions presented in the study are included in the article/**Supplementary Material**. Further inquiries can be directed to the corresponding author.

## Ethics statement

The studies involving human participants were reviewed and approved by Peking Union Medical College Hospital. The patients/participants provided their written informed consent to participate in this study.

## Author contributions

LZ and XZ contributed to the conception of the study. JN, XS and HW performed the experiment. JN contributed to analysis and manuscript preparation. JN performed the data analyses and wrote the manuscript. All authors contributed to the article and approved the submitted version.

## Supplementary material

Supplementary Figure 1 Baseline characteristics of patients by treatment pattern.

Supplementary Figure 2 Forest plot of subgroup analysis of progression-free survival.
