# Supplementary figures and images for: Camrelizumab plus platinum-irinotecan followed by maintenance camrelizumab plus apatinib in untreated extensive-stage small-cell lung cancer: a nonrandomized clinical trial
Source: Front Immunol. 2023 Apr 11;14:1168879. doi: 10.3389/fimmu.2023.1168879 (PMC10126331; doi:10.3389/fimmu.2023.1168879)

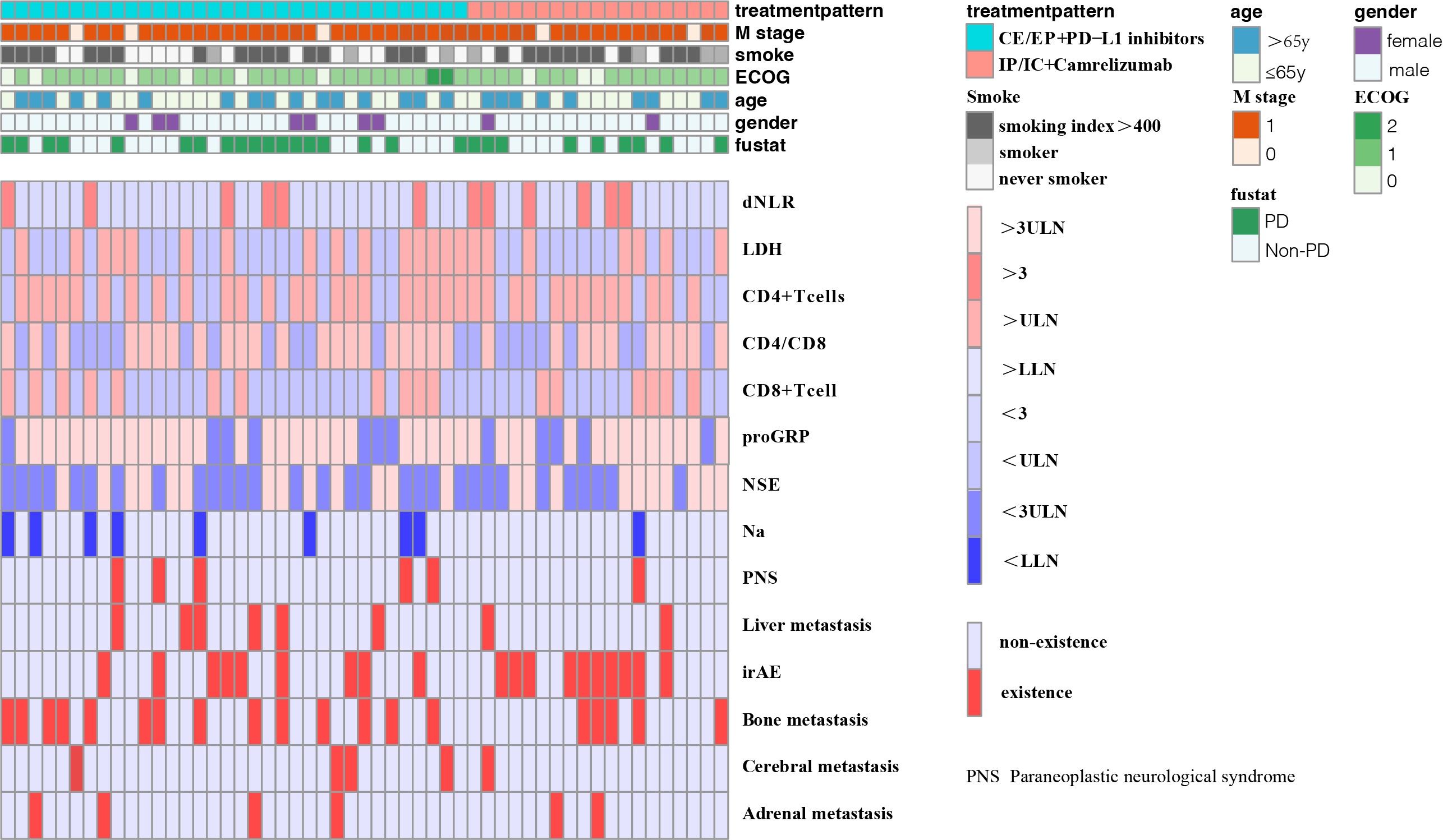

Supplement: Supplementary Figure 1 — Baseline characteristics of patients by treatment pattern. [file Image_1.jpeg]

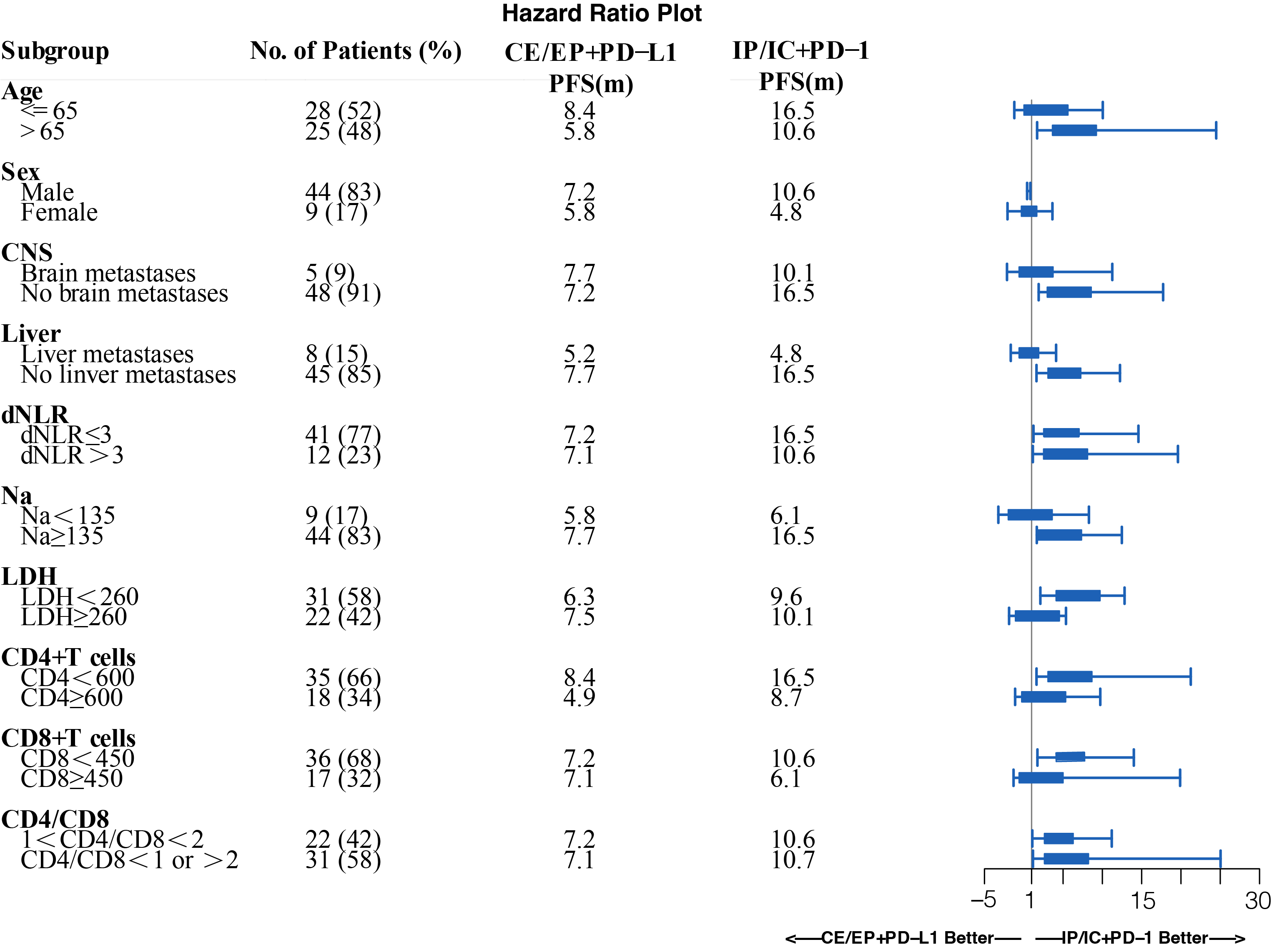

Supplement: Supplementary Figure 2 — Forest plot of subgroup analysis of progression-free survival. [file Image_2.jpeg]
