# Supplementary material for: Camrelizumab plus platinum-irinotecan followed by maintenance camrelizumab plus apatinib in untreated extensive-stage small-cell lung cancer: a nonrandomized clinical trial
Source: Front Immunol. 2023 Apr 11;14:1168879. doi: 10.3389/fimmu.2023.1168879 (PMC10126331; doi:10.3389/fimmu.2023.1168879)
Supplement: Supplementary file 3 [file Table_1.docx]

Inclusion Criteria

1. Aged 18 to 75 years old.
2. Extensive small cell lung cancer diagnosed by pathology or cytology.
3. Patients with extensive stage small cell lung cancer who have not previously been treated for tumors (including radiotherapy, chemotherapy, use of similar VEGFR inhibitors and immune checkpoint inhibitors).
4. The disease progress of the patients with limited SCLC who had received chemotherapy/radiotherapy before, and no further treatment more than 6 months after the last chemotherapy/radiotherapy.
5. Expected survival more than 3months.
6. Eastern Cooperative Oncology Group (ECOG) performance status 0-1.
7. Subjects enrolled must have measurable lesion(s) according to the RECIST 1.1 standard (the CT scan length of the tumor lesion > 10 mm)
8. The main organ function is normal. All baseline laboratory requirements will be assessed and should be obtained within -14 days of randomization. Screening laboratory values must meet the following criteria. A) Hemoglobin ≥ 9.0 g/dL (90 g/L); B) Absolute neutrophil count ≥ 1.5 x 10^9^/L; C) Platelets ≥ 100 x 10^9^/L; D) Total bilirubin (TBIL) ≤ 1 x upper limit of normal (ULN); E) Alanine aminotransferase (ALT) and aspartate aminotransferase (AST) ≤ 1.5 x upper limit of normal (ULN); alkaline phosphatase ≤ 5 x ULN; F) Serum creatinine ≤ 1.5 x ULN or creatinine clearance > 45 mL/minute (using Cockcroft/Gault formula)
9. Female participants of childbearing potential must have a negative serum pregnancy test within -7 days of randomization and must be willing to use efficient barrier methods of contraception or a barrier method plus a hormonal method starting with the screening visit through 60 days (about 5 drug half-lives + menstrual cycle) after the last dose of camrelizumab. Male participants with a female partner(s) of child-bearing potential must be willing to use efficient barrier methods of contraception from screening through 120 days (about 5 drug half-lives + sperm depletion cycle) after the last dose of camrelizumab.
10. Subjects should voluntarily participate in clinical studies and informed consent should be signed.

Exclusion Criteria:

1. Imaging (CT or MRI) showed the presence of a central tumor that invades the local large vessels. Or there are obvious pulmonary cavitation or necrotizing tumors.
2. Patients with active brain metastasis or meningeal metastasis.
3. Subjects used immunosuppressive drugs excluding nasal spray and inhaled corticosteroids or systemic steroids at physiological doses (prednisolone≤10 mg/day or other corticosteroids of the same pharmacophysiological dose) within 14 days before the first dose.
4. Uncontrollable hypertension (systolic blood pressure ≥140 mmHg or diastolic blood pressure ≥90 mmHg, despite with the optimal medical treatment).
5. Subjects with grade II or above myocardial ischemia or myocardial infarction and poorly controlled arrhythmias (QTc interval > 450 ms for males and QTc interval > 470 ms for females). Subjects with grade III-IV cardiac insufficiency or with left ventricular ejection fraction (LVEF) less than 50% had myocardial infarction within 6 months before admission according to NYHA criteria.
6. Accompanied by uncontrolled pleural effusion, pericardial effusion, or ascites requiring repeated drainage.
7. Participants who had any active autoimmune disease or a history of autoimmune disease (such as the following, but not limited to: autoimmune hepatitis, interstitial pneumonia, uveitis, enteritis, hepatitis, pituitary inflammation, vasculitis, nephritis, hyperthyroidism, decreased thyroid function).
8. Subjects with childhood asthma has completely resolved, adults can be included without any intervention; subjects with bronchodilators for medical intervention cannot be included.
9. Participants who had abnormal blood coagulation (INR>1.5 or PT> ULN+4s, and or APTT > 1.5 ULN), bleeding tendency or receiving thrombolytic or anticoagulation；Note: under the premise that the international standardized ratio of prothrombin time (INR) is ≤ 1.5, the use of low-dose heparin (60,000-12,000u per day for adults) or low-dose aspirin (≤ 100mg per day) is allowed for preventive purposes.
10. Urine routine indicates urinary protein ≥ ++, or confirms that 24-hour urine protein is ≥1.0 g.
11. Patients with non-healing wound, non-healing ulcer, or non-healing bone fracture.
12. The patient has severe infection within 4 weeks before first administration（such as the need for intravenous antibiotics, antifungals or antivirals) and unexplained fever within 7 days before administration, ≥38.5 °.
13. There was significant coughing blood and significant clinically significant bleeding symptoms or a clear tendency to hemorrhage in the first 2 months before enrollment (such as gastrointestinal bleeding, hemorrhagic gastric ulcer, fecal occult blood ++ and above at baseline, or suffering from vasculitis, etc.).
14. Serious arterial/venous thrombosis events, such as cerebrovascular accidents (including transient ischemic attacks, cerebral hemorrhage, cerebral infarction), deep vein thrombosis, and pulmonary embolism, within 12 months before enrollment.
15. Known history of testing positive for human immunodeficiency virus (HIV) or known acquired immunodeficiency syndrome (AIDS). Positive test for hepatitis B virus surface antigen (HBV sAg) or hepatitis C virus ribonucleic acid (HCV RNA) indicating acute or chronic infection. (HBV: HBsAg positive and HBV DNA ≥ 500 IU/mL; HVC: HCV RNA positive and abnormal liver function). And subjects with active tuberculosis.
16. Patients with a clear history of allergies may be potentially allergic to or intolerant to biological agents such as irinotecan, cisplatin, apatinib, and camrelizumab.
17. There are obvious factors affecting oral drug absorption, such as inability to swallow, chronic diarrhea and intestinal obstruction. Or sinus or perforation of empty organs within 6 months.
18. Any known mental illness or substance abuse that may have an impact on compliance with the test requirements.
19. There are other factors lead to patients unable to participate in this clinical study by the judgment of the investigator.
